# Orosomucoid, a New Biomarker in the Association between Obesity and Periodontitis

**DOI:** 10.1371/journal.pone.0057645

**Published:** 2013-03-19

**Authors:** Hélène Rangé, Christine Poitou, Adrien Boillot, Cécile Ciangura, Sandrine Katsahian, Jean-Marc Lacorte, Sébastien Czernichow, Olivier Meilhac, Philippe Bouchard, Catherine Chaussain

**Affiliations:** 1 Department of Periodontology, Service of Odontology, Rothschild Hospital, AP-HP, Paris 7-Denis Diderot University, U.F.R. of Odontology, Paris, France; 2 INSERM U698, Cardiovascular Hematology, Bioengineering and Remodeling, Bichat Hospital, AP-HP, Paris 7-Denis Diderot University, Paris, France; 3 Nutrition and Endocrinology Department, Pitié-Salpêtrière Hospital, AP-HP, Paris, France; 4 CRNH-Ile de France, Paris, France; 5 Institute of Cardiometabolism and Nutrition (ICAN), Paris, France; 6 INSERM U872, Team 7 Nutriomics, Cordeliers Research Center, Paris, France; 7 Paris 6-Pierre et Marie Curie University, UMR S 872, Paris, France; 8 INSERM U1018, Centre for Research in Epidemiology and Population Health, Villejuif, France; 9 Department of Biostatistics, Saint Louis Hospital, AP-HP, Paris 7-Denis Diderot University, Paris, France; 10 Department of Endocrinology and Oncology Biochemistry, Pitié-Salpêtrière Hospital, AP-HP, Paris, France; 11 Department of Nutrition, Ambroise Paré Hospital, AP-HP, Versailles St-Quentin University, Boulogne-Billancourt, France; 12 Service of Odontology, Bretonneau Hospital, AP-HP, Paris 5-Descartes University, EA 2496, U.F.R. of Odontology, Paris, France; Universidad Pablo de Olavide, Centro Andaluz de Biología del Desarrollo-CSIC, Spain

## Abstract

Epidemiological data indicate an association between periodontitis and obesity. The biological mechanisms of this relationship remain unclear. A cross-sectional study was conducted to evaluate the relationship between periodontitis and the common systemic inflammatory markers in 32 morbidly obese patients recruited in a Clinical Nutrition department. Periodontal condition was evaluated using pocket depth (PD) measurement, a classical clinical marker of ongoing periodontitis. Major periodontal risk factors were recorded (age, gender, diabetes and smoking status), as well as plasma levels of inflammatory markers (CRP, orosomucoid, IL-6) and adipokines (adiponectin, leptin). All patients included in the sample exhibited evidence of periodontitis, 16 of whom were diagnosed as having severe disease. Adjusted logistic regression analysis indicated that the severity of periodontitis was associated with the plasma level of orosomucoid (p<0.04) after adjustment for age, gender and smoking. Our study thus suggests that the severity of periodontitis, in morbidly obese patients, is associated with the increase of orosomucoid levels.

## Introduction

Periodontitis comprises a group of multifactorial inflammatory diseases that affect the periodontium, i.e. the epithelial, connective and bone tissues that both surround and support the teeth [1]. From a pathophysiological point of view, inflammatory host mediators are involved in the detachment of the gingival connective tissue from the root surface, and in the resorption of alveolar bone supporting the tooth. The natural history of the disease leads to tooth loss. Chronic periodontitis is one of the most prevalent low-grade, bacterially induced, chronic inflammatory diseases affecting 20 to 50% of the adult population worldwide [2], [3]. The low-grade inflammation associated with chronic periodontitis is characterized by increased levels of circulating pro-inflammatory cytokines (IL-1, IL-6, tumor necrosis factor α) and C-reactive protein [4], [5]. Similarly, low-grade inflammation is the hallmark characterizing adult obesity, with increased levels of plasma inflammatory markers (C-reactive protein, IL-6, serum amyloid A, fibrinogen and orosomucoid) and changes in adipokines (adiponectin, leptin). All these markers are possibly involved in obesity-related comorbidities such as type 2 diabetes and atherosclerosis [6], [7].

Numerous data indicate the impact of periodontal diseases on health [8]. An increased prevalence of diabetes, rheumatoid arthritis, atherosclerosis, myocardial infarction and stroke has been reported in patients with periodontal disease [9], [10], [11]. The underlying biological mechanism involves local periodontal inflammation that may increase the levels of systemic inflammatory mediators, thereby promoting atherosclerosis and insulin resistance [12]. A potential link between obesity and periodontitis has also been shown [13], [14], [15]. Obesity may be a factor contributing to periodontitis severity via a modulation of the immune system [16]. However, little is known about the systemic effects of periodontitis on obesity and its related comorbidities [17], [18], [19]. To date, no study has specifically focused on the periodontal status of morbidly obese subjects (Body Mass Index ≥40 kg/m^2^). We hypothesized that periodontitis in morbidly obese subjects could alter the profile of inflammatory mediators. Consequently, we conducted a study to determine the extent to which periodontitis influences systemic levels of inflammatory mediators in a group of morbidly obese patients.

## Methods and Procedures

### Selection of subjects

In this cross-sectional study, all included subjects were recruited from the patients referred to the Department of Nutrition, Center of Reference for Medical and Surgical Care of Obesity (CREMO, Pitié-Salpêtrière hospital, Paris, France) for bariatric surgery. Before surgery, these patients underwent a periodontal screening at the Department of Odontology, Bretonneau Hospital (Paris, France). All patients, referred between September 2007 and July 2008, were considered for inclusion. Body weight was measured to the nearest 0.1 kg with subjects in indoor clothing and no shoes. Height was measured to the nearest 0.5 cm with a wall-mounted stadiometer, in the same conditions. The subject's weight was stable (i.e. variation of less than ±2 kg) for at least 3 months prior to the operation. Subjects did not demonstrate evidence of acute or chronic inflammatory disease, infectious diseases, viral infection, cancer and/or known alcohol consumption (>20 g per day). Patients having rheumatoid arthritis, malignant disease, or a past history of cardiovascular disease were excluded from the study. Patients were considered type 2 diabetics if they used an oral antidiabetic treatment, or had fasting blood glucose ≥1.26 g/l or glycated hemoglobin above 6.5%. Included patients had to have 10 or more teeth. Smoking status (current, former, and never) was evaluated quantitatively as the number of cigarettes per day. The study protocol was approved by the Ethics Committee of Paris Ile-de-France, and all the participants provided their written, informed consent to participate in the study.

### Periodontal examination

All the examinations were completed by one periodontist (H.R.), who was calibrated for probing to a “gold standard” senior clinical researcher (P.B.) before the study. Examiner calibration was considered effective for an intraclass correlation coefficient ≥0.9.

The following classical parameters were recorded:

*Number of teeth* – number of teeth, excluding third molars, which remained in the mouth.

*Quantity of Dental plaque*–the Plaque Index score system (PI) [20] was used to assess the thickness of plaque at the cervical margin of the tooth (closest to the gum). Each tooth was dried and examined visually using a mirror, a probe, and adequate light. The probe was passed over the cervical third to test for the presence of plaque. A disclosing agent may have been used to assist evaluation (Dento-Palque® Inava, Pierre Fabre Oral Care, France). Four different scores were possible. A zero indicated no plaque present on the tooth; 1 indicated a film of plaque observable only after application of disclosing solution or by using the probe on the tooth surface; 2 represented moderate accumulation of soft deposits in the gingival pocket or on the tooth that could be seen by the naked eye; 3 represented an abundance of soft matter within the pocket or on the tooth. Thus, each area of each tooth was assigned a score from 0 to 3. Scores for each tooth were totaled and divided by the six surfaces scored. To determine a median PI for an individual, the scores for each tooth were added and divided by the number of teeth examined. Four ratings could then be assigned: 0 = excellent, 0.1–0.9 = good, 1.0–1.9 = fair, 2.0–3.0 = poor. A PI≥1.0 was the threshold for qualifying plaque control as insufficient.

*Gingival inflammation* – the Gingival Index score system [GI] [21] was used to assess the severity of gingivitis based on color, consistency, and bleeding on probing. Each tooth was examined at six sites. A probe was used to press on the gingiva to determine its degree of firmness, and to run along the soft tissue wall adjacent to the entrance to the gingival sulcus. Four criteria were possible: 0, normal gingiva; 1, mild inflammation but no bleeding on probing; 2, moderate inflammation and bleeding on probing; 3, severe inflammation and ulceration, with a tendency for spontaneous bleeding. In our study, each surface was given a score, and then the scores were totaled and divided by six. That number was divided by the number of teeth examined to determine the median GI. Ratings were 0 = excellent; 0.1–1.0 = good; 1.1–2.0 = fair; 2.1–3.0 = poor. A GI>1.0 was the threshold for diagnosing gingivitis.

*Periodontal disease*–the Pocket Depth (PD) was recorded in millimeters from the gingival margin to the bottom of the pocket using a manual periodontal probe (HuFriedy PCP UNC 15, Chicago, IL, USA). Measurements were taken to the nearest millimeter at 6 sites around each tooth.

*Periodontal destruction*–the Attachment Level (AL) was calculated in millimeters by adding the pocket depth value and the gingival recession value.

*Case definition*–Periodontitis was defined as a disease state in which there was an active destruction of the periodontal tissues as evidenced by the simultaneous presence of ≥3 mm pocket depth (PD), ≥2 mm attachment level (AL) and bleeding on probing (GI>2) at least 2 sites on 2 non-adjacent teeth [22]. Severe periodontitis was defined as at least 2 sites on 2 non-adjacent teeth with probing depth ≥5 mm and bleeding on probing (GI>2).

### Inflammatory mediator quantitation

Venous blood samples were collected in the fasting state for routine determination of several biochemical parameters outlined in [6], [23]. Serum samples were stored at −80°C before assessing other biological parameters, including levels of leptin, adiponectin, orosomucoid and acute phase response markers (CRP, IL-6). Serum leptin and adiponectin were determined using a radio-immunoassay kit from Linco research (Saint Louis, MI, USA) according to the manufacturer's recommendations. The sensitivity was 0.5 ng/ml and 0.8 µg/ml for leptin and adiponectin respectively. Intra-assay and inter-assay coefficients of variation (CVs) were below 4 and 9% for leptin and adiponectin respectively. Serum levels of IL-6 were measured by a high-sensitivity ELISA system (Quantikine HS, R&D System Europe Ltd., UK). The sensitivity of this assay was <0.04 pg/ml and intra-assay and inter-assay CVs were below 8%. High sensitivity CRP (hsCRP) and orosomucoid levels were measured with an IMMAGE automatic immunoassay system (Beckman–Coulter, Fullerton, California, USA) of sensitivity 0.02 and 35 mg/dl, respectively; intra-and interassay CVs were <5% and 7.5%, respectively, for hsCRP and 4% and 6% for orosomucoid.

### Statistical Analysis

All analyses were performed using the statistical software R (R, version 2.12.1, the R Core Development team, 2010). *A priori* sample size calculation was performed using a statistical software program [24]. Using the patient as the statistical unit and hsCRP value as the main variable, a sample size of 32 was calculated to achieve 80% power at the two-sided 5% level to detect a difference of 4 mg/L between the null hypothesis and the alternative hypothesis, with a standard deviation of 4 mg/L.

The population was separated into two groups: patients with mild to moderate periodontitis (n = 16); and those with severe periodontitis (n = 16). Differences in clinical and demographic characteristics between groups were analysed using the Wilcoxon rank sum test and the Fisher exact test (Table 1). First, the univariate model was run to explore the association between severity of periodontitis and biological (CRP, orosomucoid, Il-6, adiponectin and leptin) and non-biological (number of teeth, BMI, diabetes and smokers) variables (Table 2). Then, all biological variables were included in the multivariate models with adjustment for age, gender and smoking (Model A) and with adjustment for age, gender, smoking and diabetes (Model B) (Table 3).

**Table 1:** Bioclinical and periodontal characteristics of the population studied.

Parameters (units)	Mild to moderate Periodontitis (n = 16)		Severe Periodontitis (n = 16)		Total (n = 32)	
	*Median*	*range*	*median*	*range*	*median*	*range*
Age (years)	45.5	31.0–60.0	46.0	34.0–60.0	46.0	31.0–60.0
BMI (kg/m^2^)	48.1	37.0–73.5	47.5	36.3–60.9	47.5	36.3–73.6
Females *n* (%)	13 (81)	-	12 (75)	-	25 (78)	-
Diabetes *n* (%)	8 (50)	-	9 (56)	-	17 (53)	-
Smokers *n* (%)(1)	10 (62)	-	5 (41)	-	15 (47)	-
Remaining teeth *n*	27	10–28	26	11–28	26	10–28
PI	1.0	0.3–2.8	1.1	0.4–2.2	1.0	0.3–2.8
GI	1.8	1.4–2.9	2.1	1.0–2.7	1.9	1.0–2.9
PPD (mm)*^†^*	2.5	1.8–2.7	2.8	2.4–4.5	2.6	1.8–4.5
CAL (mm)*^†^*	2.6	1.8–4.3	2.9	2.4–5.0	2.8	1.8–5.0
CRP (mg/l)	5.0	1.0–23.8	6.2	1.5–62.8	5.6	1.0–62.8
Orosomucoid (g/l)^*^	0.9	0.6–1.2	1.1	0.6–1.3	1.0	0.6–1.3
Il–6 (pg/ml)	3.1	1.5–28.0	3.6	1.8–11.5	3.3	1.5–28.0
Adiponectin ( µg/ml)	7.7	3.1–11.7	6.5	3.1–10.9	7.4	3.1–11.7
Leptin (ng/ml)	46.9	15.4–85.0	44.3	22.7–98.3	45.6	15.4–98.3

The Wilcoxon rank sum test was used to compare medians between groups, and the Fisher exact test to compare proportions. *p<0.05. ^†^p<0.01. PI: Plaque Index, GI: Gingival Index, PPD: Pocket Probing Depth, CAL: Clinical Attachment Loss, CRP: C-Reactive Protein.

(1) Smoking status: never versus former and current.

**Table 2:** Association between non-biological and biological variables and severity of periodontitis (Severe Periodontitis versus Mild to moderate Periodontitis) (p values from univariate models).

Parameters (units)	Severe Periodontitis versus Mild to moderate Periodontitis	
	p value	OR [95% CI]
Number of remaining teeth	0.84	1.01 [0.88; 1.17]
BMI (kg/m^2^)	0.88	0.99 [0.91; 1.08]
Diabetes *n* (%)	0.72	1.29 [0.32; 5.30]
Smokers *n* (%)(1)	0.08	0.27 [0.06; 1.13]
CRP (mg/l)	0.40	1.04 [0.97; 1.16]
Orosomucoid (mg/dl)	0.09	1.05 [0.99; 1.11]
Il 6 (pg/ml)	0.66	0.97 [0.77; 1.13]
Adiponectin ( µg/ml)	0.50	0.92 [0.71; 1.17]
Leptin (ng/ml)	0.57	1.00 [0.99; 1.00]

(1) Smoking status: never versus former and current, OR: Odds Ratio, CI: Confidence Interval.

**Table 3:** Adjusted logistic regression models for the association of severity of periodontitis and concentrations of inflammatory biomarkers.

Parameters (units)	Model A		Model B	
	OR [95%CI]	p value	OR [95%CI]	p value
CRP (mg/l)	1.04 [0.96; 1.19]	0.44	1.05 [0.97; 1.20]	0.37
Orosomucoid (mg/dl)	1.06 [1.00; 1.14]	0.04*	1.06 [1.00; 1.14]	0.053
Il 6 (pg/ml)	0.99 [0.78; 1.17]	0.90	0.99 [0.79; 1.18]	0.93
Adiponectin ( µg/ml)	0.93 [0.67; 1.28]	0.67	0.87 [0.60; 1.22]	0.44
Leptin (ng/ml)	1.00 [0.97; 1.05]	0.65	1.00 [0.96; 1.05]	0.86

The Model A is adjusted for age, gender and smoking (never versus former and current) and the Model B is adjusted for age, gender, smoking and diabetes (n = 32).

* p<0.05, OR: Odds Ratio, CI: Confidence Interval.

## Results

### Periodontal status of obese patients

Thirty-two subjects were included in the analysis. Table 1 shows the bioclinical characteristics of the obese subjects. The median (range) age of the sample was 46.0 (31.0–60.0), and females were over-represented, accounting for 78% of the sample (n = 25). Fifty three percent of patients were diabetic, and 47% were smokers. CRP levels were high in agreement with obesity-associated low-grade inflammation. The results [median (range)] indicate that the obese subjects had lost teeth i.e. number of remaining teeth = 26 (10–28), had dental plaque accumulation i.e. Plaque Index = 1.0 (0.3–2.8), had gingivitis i.e. Gingival Index = 1.9 (1.0–2.0) and had periodontal attachment loss i.e. Clinical Attachment Loss = 2.8 (1.8–5.0) (Table 1). A median value of PI>1, indicates a moderate accumulation of dental plaque and a median value of 1<GI≤2, indicates moderate inflammation. Increased PI and increased GI have been shown to be associated with an increased risk of periodontal disease. The 32 morbidly obese subjects included had mild to moderate (n = 16) or severe chronic periodontitis (n = 16). The periodontal diagnosis was based on the Armitage classification of periodontal diseases [25]. A significant difference was observed between the mild to moderate and severe periodontitis patients for median pocket depth (p<0.01) and median attachment loss (p<0.01). The percentage of diabetes was not different between the groups with mild to moderate and severe periodontitis (50% vs 56%). The percentage of non-smokers in the group of mild to moderate periodontitis was double that in the group with severe disease (38% vs 69%), without reaching statistical significance.

### Orosomucoid concentration is associated with periodontitis severity

Median (range) orosomucoid concentration was lower in the mild to moderate periodontitis group compared to the severe periodontitis group 0.9 (0.6–1.2) vs 1.1 (0.6–1.3) respectively, p<0.05) (Table 1). The results of the univariate analysis of the association between the severity of periodontitis and the non-biological and biological variables are presented in Table 2. Table 3 indicates that severe periodontitis status was positively associated with the plasma levels of orosomucoid (p = 0.04) in the adjusted logistic regression model A (with adjustment for age, gender and smoking) but without reaching statistical significance (p = 0.053) in Model B (with adjustment for age, gender, smoking and diabetes).

## Discussion

The data of the present study indicate that morbidly obese patients are prone to chronic periodontitis, and that disease severity is significantly associated with the circulating concentration of orosomucoid.

Regarding the poor periodontal condition in obese patients, our results are consistent with others showing that poor oral health is related to high BMI. Two meta-analyses have shown an association between BMI and periodontitis, although the magnitude of the relationship remains unclear [13], [15]. In an adult French population, it has been found that BMI was statistically associated with missing teeth, pocket depth and plaque index, independently of dietary patterns and insulin resistance [26]. Our results indicate a median missing tooth value of 4, which is similar to the median number of missing teeth in the general French population i.e. 3.8 (95% confidence interval: 1.6–7.6) [27]. Nevertheless, it has been demonstrated that the chewing patterns were affected in patients with morbid obesity as compared with controls, whatever the number of teeth present [28]. Consequently, it cannot be excluded that periodontitis, and not edentulism, negatively impacts the chewing ability and the quality of life of morbidly obese subjects. On the other hand, in the present study the Gingival Index, a clinical marker of local periodontal inflammation, did not significantly differ between mild to moderate periodontitis and severe periodontitis patients. It cannot be excluded that low grade inflammation associated with obesity obscures the clinical expression of local inflammation in the development of periodontitis. A recent study using a proteomic approach has shown an increased level of antimicrobial peptides (defensins) in the saliva of morbidly obese patients suffering from periodontitis [29]. A two-way relationship between obesity-induced and periodontitis-induced inflammation cannot be ruled out.

Moreover, among the various adipokines and inflammatory markers studied here, only the orosomucoid level was associated with periodontal inflammation severity after adjustment for age, gender and smoking. In a comparative study on systemic inflammation in cardiovascular and periodontal disease, higher levels of orosomucoid were observed in subjects with both these conditions [30] compared to subjects with neither disease, or with only PD or CVD. Indeed, an increased level of orosomucoid is characteristic of subjects with both cardiovascular and periodontal diseases. Orosomucoid or Alpha (1)-acid glycoprotein is an inflammation-sensitive plasma protein. This protein is a typical marker of inflammation, which increases by a factor of 3–4 after an inflammatory stimulus [31], [32]. The synthesis of orosomucoid takes place in the liver and is induced by IL-1, TNFα and IL-6. Neutrophils and monocytes can also synthesize orosomucoid and thus contribute to the elevation of this protein in the serum of patients with sepsis. Interestingly, C reactive protein is an acute phase inflammatory marker whereas orosomucoid is a chronic phase marker [33], [34]. The combined assessment of CRP and orosomucoid profiles can be used to diagnose, date and monitor the inflammatory syndrome: in an early onset reaction, only CRP increases; then both CRP and orosomucoid increase; and after a successful treatment, CRP decreases first. Since periodontitis is a chronic disease of infectious origin, orosomucoid appears to be a better inflammatory marker than CRP in the morbidly obese, who display a chronic increase in CRP. Moreover, in obese patients, IL-6 synthesis is more the reflection of adipose tissue production itself than representative of systemic inflammation [35]. Therefore, IL-6 levels may correlate less with periodontal disease than CRP or orosomucoid. Indeed, the data of the present study do not show any association between CRP, IL-6, leptin or adiponectin with periodontitis in morbidly obese patients. This is in accordance with a case control study showing that periodontitis was not associated with decreased levels of adiponectin [36]. In this study, the association between severity of periodontitis and the orosomucoid levels failed to reach statistical significance after adjustment for diabetes (Table 3). A large cohort study has shown that orosomucoid levels were associated with the development of diabetes mellitus in middle-aged adults [37]. Recently, an animal study has suggested that orosomucoid may be involved in the reduction of neutrophil migration and increased susceptibility to sepsis in diabetic mice [38]. Diabetes could thus introduce a bias in the association between high orosomucoid levels and periodontitis in obese patients with impaired neutrophil function.

Furthermore, orosomucoid is a marker of malnutrition. There is a link between nutritional status and periodontitis [39] which could explain the association between orosomucoid level and pocket depth in morbidly obese patients. Nevertheless, the orosomucoid threshold to define malnutrition is 1.2 g/l and in this study none of the morbidly obese patients had orosomucoid plasma levels reaching that value.

The present study has several strengths. First, it is the first study to deal with the association between periodontal parameters and systemic inflammation markers in a morbidly obese population. We chose morbid obesity as a model because it represents the most extreme condition of obese patients, i.e. the worst inflammatory condition. To our knowledge, this is the first clinical study demonstrating a systemic link among obesity, periodontitis, and systemic low-grade inflammation. Second, we do not use a specific classification for periodontal diseases. Instead, we use clinical periodontal parameters to enable comparisons with other available data. The definition of periodontitis varies considerably in epidemiological studies [22]. The use of a diagnostic classification may jeopardize the analysis by increasing the risk of finding spurious associations. Third, we included a full-mouth periodontal clinical examination at six sites per tooth, to obtain a detailed periodontal status of all individuals. Therefore, the sensitivity of our study may be higher than others which used partial recording and/or self-reported data.

However, this study has limitations inherent to its cross-sectional design that cannot determine a causal relationship. It may also be assumed that our sample size is relatively small. Even if an increase in morbid obesity prevalence is observed in industrialized countries, the global prevalence of this special patient category is fortunately still low, i.e. in the USA 2.8% of men and 6.9% of women are affected [40] and especially in France, where it affects 1.1% of all adults [41]. Moreover among this special patient category, few are scheduled for bariatric surgery. Consequently, the number of subjects able to be included in the study was limited. The limited sample size could explain that no differences in the levels of adipokines and inflammatory mediators reached statistical significance between obese patients with severe periodontitis and those with mild to moderate disease. Last, the impact of socioeconomic status respectively on obesity [42] and on periodontitis [43] is now well documented, and we cannot exclude the possibility that socioeconomic inequalities could influence periodontitis susceptibility in obese subjects.

The conclusions of our study support the hypothesis that localized persistent infection may influence systemic levels of inflammatory mediators. Periodontal infection could aggravate the inflammatory state of the morbidly obese patient by increasing the plasma levels of orosomucoid and contribute to the development of obesity-related morbidity, such as atherosclerosis [44]. More evidence is required to evaluate the association between periodontal diseases, obesity and cardiovascular diseases. Since this study should be considered as preliminary, the consistency of the association might be explored in other clinical studies monitoring the common inflammatory mediators (CRP, Il 6, adiponectin, leptin), including orosomucoid, in obese patients and in non-obese controls with and without diabetes. In preventive clinical practice, a comprehensive periodontal and dental examination could be included in the follow-up of morbidly obese patients.

## Acknowledgments

The authors thank Pr Arnaud Basdevant for his advice in the manuscript revision and Dr Florence Marchelli and Patricia Ancel who were involved in data collection and sampling at the Center for Research on Human Nutrition, Pitié-Salpêtrière Hospital, Paris. We are also grateful to Dr Mary Osborne-Pellegrin (INSERM U698, Bichat Hospital, Paris) for help in editing the manuscript.
